# Some Notes on Medical and Surgical Experiences in Cuba*Read before the Orleans Parish Medical Society, December meeting, 1898.

**Published:** 1899-02

**Authors:** Hamilton P. Jones

**Affiliations:** Late Acting Assistant Surgeon United States Army, Clinical Assistant New Orleans Polyclinic, Visiting Surgeon Charity Hospital. New Orleans; Assistant Demonstrator on Chemistry, Medical Department, Tulane University of Louisiana, New Orleans


### SOME NOTES ON MEDICAL AND SURGICAL EXPERIENCES IN CUBA.*

BY DR. HAMILTON P. JONES.

Late Acting Assistant Surgeon United States Army, Clinical Assistant New Orleans Polyclinic, Visiting Surgeon Charity Hospital, New Orleans; Assistant Demonstrator on Chemistry, Medical Department, Tulane University of Louisiana, New Orleans.

It was suggested to me by the chairman of the Scientific Committee that I give an account of the conditions prevailing in Cuba during the recent campaign, both surgical and medical.

I shall confine myself to my personal experiences and will present a simple narrative account of them.

On the fourth day of May I entered active army life with the Eleventh United States Infantry, at Mobile, Ala., with whom I remained almost a week. This camp was located back of Mobile in the high piney woods and was a well ordered and healthy camp, laid out and kept policed and clean, according to the regulations of the United States Army. Here the surgeon of the regiment inspected the sinks and cook tents twice a day, accompanied by the officer of the day, to whom all suggestions and complaints regarding the sanitary condition were made, and whose duty it was to see that the suggestions were carried out and the causes for complaint abated. Should the officer of the day fail through neglect to do his duty to the surgeon, recourse was at once had to the colonel of the regiment—a contingency which I never saw arise. I go thus fully into this matter because the same conditions prevailed at Tampa Heights, just outside of Tampa. Both these camp sites were similar in that they were in the piney woods just outside of and were elevated above the general level of rather large cities; and more than this, each one had a comparatively wholesome supply of drinking water, which could not be readily contaminated by fecal matter. These camps were both exceedingly healthy up to the time of my going to Cuba. I remained nearly a month at Tampa.

On June 1st, I was ordered to the First Division Hospital, which was organized under the command of Maj. Marcus W. Wood, General Kent's chief surgeon, with Maj. R. W. Johnson in charge, Lieut. Guy M. Godfrey in command of the hospital corps, Dr. Frederick J. Combe and myself on the staff.

The equipment for this hospital was procured by stripping the regimental hospitals of almost everything, a difficult task, as they had already been twice subjected to this ordeal, for the formation of the other two division hospitals. Operating tables and the like were procured without much difficulty from the medical supply depot at Tampa. Certain articles, such as blue-flame oil-burners for sterilization purposes, zinc tanks,

*Read before the Orleans Parish Medical Society, December meeting, 1898.

twenty gallons capacity, for holding sterilized water, small size round bar iron in eight-foot lengths for the manufacture of splints, etc., were either ordered or purchased in Tampa. About twenty-five hospital tents and about fifty officers' tents, all complete, were secured, and an ambulance train consisting of twenty perfectly new ambulances with new harness and four good mules each and driver, extra mules and an abnndant supply of forage and grain and a competent Western trainmaster were gotten together and organized. In spite of the shortness of time given for the organization of this hospital and its equipment, a little over five days, it was in a remarkable state of fitness for the work which was subsequently thrust upon it. Had we been allowed to proceed to Cuba prepared as we were, an infinite amount of suffering, labor and criticism would have been avoided, but the order directing us to embark *commanded us to abandon all tentage except flies and to leave behind our precious ambulances.* Had we had our ambulances in Cuba the failure to furnish us with transportation would have been a matter of not the slightest importance. But the loss of our ambulances and the almost entire absence of any other form of transportation caused suffering, misery and death, the extent of which can only be appreciated by those who endured and those who witnessed it. Out of forty ambulance-loads of our hospital stuff on board the transport Santiago, only two six-mule army wagon-loads of the *most necessary* articles reached us in time to be of use during the fighting at El Caney, San Juan and the vicinity of Santiago on the 1st, 2d and 3d of July, and this was the only hospital in the field.

In getting their supplies aboard, the medical officers acted as stevedores, the supplies were put by themselves in the hold of the vessel, but became so disarranged that a day or two before we landed we knocked down about sixty bunks, near a port-hole on an upper deck, and piled and assorted our supplies in this cleared space, where they could be and were easily unloaded.

Except for some heat prostrations following some ill-advised practice marches made to give the men exercise while at Port Tampa, the health of the command was excellent. We learned more about heat prostrations and how to differentiate them from concussion after we reached Cuba. Our trip to Cuba was exceedingly pleasant, although rather too long, but even this was not without its pleasures, because the frequent stops from one cause and another enabled each man to bathe in the sea nearly every day.

The travel-rations furnished the men consisted practically of canned corned-beef, canned tomatoes, canned Boston baked beans, hard-tack and coffee and sugar. No lime-juice, so far as I know, was provided, although later the supply was abundant in the hospitals. I made it my duty to watch the physical condition of the well men from day to day, and particularly, as many of them had no means of looking after their teeth or the toilet of the mouth, I devoted special attention to the condition of the mouth, teeth and gums.

The diet on land consisted principally for some weeks of bacon, hard-tack, canned tomatoes, coffee and sugar.

Slight scorbutic symptoms began to show themselves to me about the 5th or 6th of July. These scorbutic symptoms never became very severe, owing to the fact that after that date the men foraged for limes and fruit. Many of the men, owing to neglect of the teeth, also suffered from gingivitis; both of which caused a condition of the gums at times very closely resembling that found in yellow fever.

I landed at Siboney on the morning of the 25th of June, after being nineteen days at sea. On the evening of the same day, acting under orders of Major Marcus Wood, General Kent's chief surgeon, I inspected the buildings in and about Siboney in order to find a suitable place for our hospital. A number of the buildings would have in a measure answered the purposes, but I reported them a menace to the health of our troops and a very probable source of yellow fever infection, and recommended their destruction. General Miles later had these same buildings burned, shortly after his arrival on the island, but not until quite a number of cases had arisen, traceable to them.

The First Division hospital left Siboney on the afternoon of the 26th.

The ambulances had been left at Tampa, as before stated, and there was no transportation at Siboney or immediate prospect of securing any, although the necessity for immediately proceeding to the front was imperative. There was no way out of it but to pack with us as much as we could and then trust to luck for the balance; for example, I was on foot and loaded with my own rations and as much chloroform, dressings, instruments and drugs as I could stagger under, leading my horse, which was also loaded with over two hundred pounds of hospital supplies. Each of the five medical officers attached to this hospital did the same thing, as did the thirty-nine hospital corps men who accompanied us, only the men in addition to their own rations and packs carried loaded litters of hospital supplies. We marched to the front by easy stages, each day going back to Siboney for more supplies, so that when we reached our final hospital site, on June 29, we had with us enough material to care for about two hundred seriously wounded men and were in advance of the entire army of invasion with the exception of a few pickets who were two hundred yards in advance on the Siboney road. We maintained this advanced position for two nights and a dav and when the troops went into action we found ourselves only 1,200 yard to the rear of the vicinity where most of the casualties occurred. On the evening of the 29th, two six-mule army wagons brought more drugs and supplies, and together with what we had packed up on our backs, served as the total supply for the only hospital in the field during that fearful, bloody time, July 1, 2 and 3, when what was to have been a reserve hospital was called upon to, and did take care of the wounded of the entire army, as they were brought from the firing line. The surgical staff of this hospital stood at the operating tables, without rest or sleep, from 8:30 A. M., July 1, to 2:30 A. M., July 3, forty-two consecutive hours, and then got up at day-break and began again.

Our hospital was established in a field a little less than three miles from Santiago, on the the Siboney-Santiago road. It lay between this road and a brook, a branch of the San Juan river. The camp was surrounded by a dense tropical jungle. The site of this camp was well chosen, and was, perhaps, as satisfactory as could have been found. We did not abandon our tentage in Tampa, but owing to lack of transportation there was tent shelter for only four operating tables and dispensary, two tents for wounded officers and wall and shelter tents for about two hundred wounded soldiers. Outside of a fair supply of instruments, operating tables and medicines and an ample supply of dressings of all sorts, our supplies were very limited. There were no cots, hammocks, mattresses, rubber blankets or pillows for sick or injured soldiers; the supply of woolen blankets was very limited, and was soon exhausted; and there was no clothing at all except a few shirts. There was no food for sick or wounded men except a few jars of beef extract, malted-milk, etc., which Major Wood brought with his private baggage and held in reserve for desperate cases. Such was the wretched equipment of the only field hospital in Cuba at the attack on Santiago, but the responsibility for its incompleteness and inadequacy can not be laid upon the field force or the medical department in Washington. We brought to the front everything we could get transportation for, and that our services and efforts in the field were recognized is attested to by the fact that General Kent recommended the five surgeons attached to this hospital for promotion and brevet.

On the 29th of July a shallow well was dug about twenty feet from the bank of the brook and above its usual daily rise caused by the afternoon rains. This insured us a clean supply of water which had percolated through and been filtered by the twenty feet of intervening sand. This water was then boiled for thirty minutes, and was finally filtered through a Berkfeldt filter, while boiling, and was placed in the zinc tanks, above mentioned, to cool. This procedure gave us a practically sterile and abundant supply of water, which, coupled with the almost universal use of the first aid packages, and the practice of modern conservatism and non-interference, gave us results unequaled in the history of field surgery.

The battle of Santiago began very early on the morning of July 1, Friday, and the wounded, most of whom had received first aid just back of the firing line, began to be received at the hospital in numbers about 9 o'cloek, and the number constantly and rapidly increased as the hot, tropical day advanced, until at nightfall long rows of wounded were lying on the grass in front of the operating tents, awaiting examination and treatment, and this in spite of our incessant work at the operating tables. As George Kennan truthfully says, we were "completely overwhelmed by the great bloody wave of human agony that rolled back in ever-increasing volume from the battle line." Notwithstanding the fact that we were reinforced during the night by surgeons from the front, the morning of the 2d found a long line of 250 men, seriously or dangerously wounded, starved, half-clothed; many of them had been lying there for hours, exposed to the chilly night air, and many more to lie for hours longer exposed to the fierce scorching sun, thirsty and hungry, and without protection from the heat. From that long row of suffering, wounded men no word of impatience or complaint came; some were undergoing the torture of the damned, and some were dying and knew it, yet they bore it with patient, quiet fortitude and tender solicitude and sacrifice to others, a display of heroism perfect and ideal. It was their splendid courage and fortitude that made their sufferings so hard to see. I have been often asked, how could the surgeons work for so great a time without rest? Well, I ask, how could a man rest with such a sight before him; when a man with a bleeding artery chides you for putting him on the operating table before attending to his companion? There were 1,500 killed and wounded during the three days' fighting; considerably over 1,000 wounded were received and treated at this hospital, and to have done it properly there should have been at least a force of fifty surgeons and 300 hospital corps men attached to the hospital, instead of the five surgeons and thirty-nine hospital corps men with which the hospital started on the 1st. Many of the regimental surgeons who came back to us at night and worked would in the morning go back to the front and return the next night. Candles were used at night, and the often-heard whistle of a guerrilla's bullet and the near-by crack of his rifle were not pleasant to the surgeon who stood with his back to a black tropical jungle. So soon as the wounded could bear transportation they were sent to Siboney, about eight miles to the rear. Every character of wound was seen, yet the majority of them were clean, perforating wounds without any explosive effects being visible, yet in a number of cases the explosive effects were very manifest, and ofttimes there would be extensive bruising of the soft tissues and great comminution of the bones. Brain injuries, as a rule, were severe and very fatal; there was nearly always some oozing of brain substance from the wound of exit. Face and neck injuries, unless some important structure was struck, did well, and it was surprising to see how some of the bullets could get through dangerous places and apparently do no great damage. Wounds of the chest and lungs, unless immediately fatal, nearly always did well. Abdominal wounds without perforation were simple, and over 50 per cent. giving evidence of intestinal injury or severe wounding of the other abdominal organs recovered without operation. Three laparotomies were performed; all terminated fatally. None of these three reached the hospital within eighteen hours of being shot, and their condition was such that they should never have been touched. The operative technic was bad and the facilities for this work poor. An hour or two spent on a laparotomy under such conditions might cost the lives of many which otherwise might be saved. Time was too valuable to waste in any such manner. Wounds of the extremities did splendidly, as did wounds of all joints, and I have recently seen men in New York and Boston and other places with knees as good as they were before they had been shot through. Three major amputations were performed. One for a fearful shell wound of the thigh, hip-joint amputation; fatal. One of lower thigh for traumatic aneurism of popliteal and consequent gangrene. One of leg for gangrene due to an Esmarch bandage, which was used as a tourniquet and which remained on for about twelve hours. Both recovered. We ran short of splint material, but Dr. Kirkpatrick, surgeon of the Twenty-fourth United States Infantry, happily discovered that the stem of last year's leaf of the Royal palm was an almost ideal splint material, available in abundance.

In this hospital, wounded Spanish captives, Cubans and Americans were treated as wounded men, without regard to nationality. Nearly every artery was ligated, repeatedly; a few trephinings and elevations were done with success. Our asepsis seems to have been as good if not better than that obtained in the best hospitals in the United States. The soldiers suffered needlessly from the promiscuous cutting and tearing of their clothes. There were no others to replace them. On July 2, Miss Clara Barton personally established a kitchen and rendered magnificent service in comforting and feeding the sick and wounded at our hospital. The care of the wounded, who were compelled by circumstances to be placed on the ground, most of them exposed to the sun and rain, was a great source of anxiety, and the work of redressing wounds and resetting fractures, catheterizing distended bladders and the general treatment of the cases was enormous. Owing to the nature of their wounds many of the men had to be given water and food through the stomach-tube. The sterilized dressings furnished by the manufacturers were aseptic.

It would take too long to go over the character of work done at that hospital, the meeting of emergencies, the transportation of wounded and the care of them, in a paper of this kind, but I hope soon to be able to present to you on lines similar to those of this paper, an account of the work done there, and of the men, their bravery, wounds, sufferings and fortitude. While we were marching backward and forward for and with hospital supplies, the solders jeered us and called us the hospital pack train and all that, but I have had more than one poor wounded fellow tell me how little he knew when he did it. To have witnessed what I did in that hospital was an awful thing, but not so awful as it was to see almost an entire army weakened and broken-spirited through the effects of the climate, exposure and disease.

The prospect of a fight and the flush of victory kept the men in good tone, and even supported many men already beginning to show the effects of the heart and the daily rains, malaria and other diseases, and it was not long after the truce was declared that they began to fall sick in hundreds, and so rapid and general was the spread of sickness that it more than once made me laugh in my sleeve to think of our having the nerve to demand a surrender. I honestly do not believe that 45 per cent of our forces could have stood the strain of a four-hours' fight, without being as badly knocked out as though they had been shot

I remained with the First Division Hospital until the 13th of July, but on the 7th of July I was made yellow fever expert at the front by Colonel Pope, chief surgeon of the Fifth Corps, and it was on that day I saw the first cases of yellow fever I had seen in Cuba, at Major Crampton's Fever Hospital, next to General Shafter's headquarters, and across the road from the field hospital.

Although there was a truce at the time, our men, who had thoroughly entrenched themselves, were lying on their arms in the trenches just as though active hostilities were in progress. These trenches, long, narrow ditches, hastily dug, with the earth thrown on the enemy's side for protection, were, many of them, very poorly drained, owing to the nature of the ground and military necessities, and all of them became quagmires at times, owing to the almost daily downpours.

The turning up of this fresh soil, most of which had been camped on recently by the Spanish, contributed its share of malarial poison, augmented by the close proximity of large numbers of Spanish dead, buried in the near-by shallow trenches abandoned by the Spanish on July 1. Santiago refugees, laden with household goods and gods, heirlooms, old letters and papers of value, poultry, dogs, cats and yellow fever germs, began pouring through our lines toward El Caney and Siboney, where they received food and a warm, hospitable welcome. These unfortunate people were permitted to mingle freely with our men and had unlimited opportunity to, and did unwittingly, spread the yellow fever infection amongst them, although the houses were the most dangerous sources of infection.

The water-supply of our troops was drawn from small mountain streams running through the rather level valley through which we passed, and these streams were used for the laundry work and bathing of over 16,000 men, and owing to the almost daily rains and absence of sinks, for some days, were also the sewers of the men and animals of the expedition, many of whom had brought with them to Cuba the seeds of typhoid, dysentery and other diseases.

Taking all these things into consideration, as well as the fact that it was almost an absolute impossibility for the men in the trenches to get anything hot for the first few days in July, the winter clothing of the men, their almost constantly drenched condition, their inability to procure dry clothes, or to build fires by which to dry themselves, and the fearfully hot days and cold nights, it was small wonder that sickness was rife to an alarming extent.

My duties carried me into almost every regiment on the front, whither I went armed with a bottle of nitric acid and a test tube, a watch and a thermometer. At the outset of my work, Colonel Pope and my dear friend "Bunkie" (Dr. Frederick Combe) were the only men who believed in my diagnosis of yellow fever, and whose loyalty to me I shall never forget. My position was an exceedingly difficult one, requiring the utmost tact and discretion, and involved not only the breaking of many diagnoses and the very frequent calling down on my head of ill feelings and bitter discussions, but involved responsibilities so great as to almost warp my judgment and weaken my convictions. Fortunately, I was morally certain of my diagnosis, and was in that happy position where, having done my duty, I could afford to wait for the other fellow to shoulder the responsibility. I later had the melancholy pleasure of having some of the medical men who doubted my diagnosis most strenuously send me some of the worst cases I received at my hospital.

Up to the time of my taking charge of the yellew fever work specially, the patients had all been sent either directly or indirectly through Major Crampton's hospital to Siboney, irrespective of diagnosis. I was ordered to set out at once to instruct such of the regimental surgeons as were not familiar with the disease in its early diagnosis, isolation and treatment, and to pick out all cases wherever found, and to see that they were isolated and transported to the rear as far as practicable. This work was hard, physically as well as mentally, and frequently caused me to ride twenty or more miles a day.

On July 13, I established what was known as Jones' Yellow Fever Hospital, on the Siboney road; about three miles from Santiago. Here the conveyances were for the first few days of the most primitive character. The men for several days lay on the ground in their shelter-tents, which were frequently flooded by the terrific rains to such an extent that I have often seen men with high fevers lying in the water one or two inches deep. Cooking utensils were luxuries at this hospital. My main soup-pot was a stolen officer's bathtub, which he had carefully brought to the front over the end of a barrel of sea-biscuits. Good cooks were as scarce as hen's teeth, and proper food for the sick very limited. But we soon learned to make a very good line of broths out of ground strained canned baked beans and the like, which offered a variety to the poor fellows, if not nourishment. Malted milk was to be had in small quantities, and did splendidly.

At one time I had at this hospital 150 serious cases of yellow fever, one urinal, one chamber, one bed-pan and several deaths as a result of going to the sinks. Supplies of all kinds were very limited at first, and some drugs were exceedingly hard to get at all times, although it was rarely the case that it was not possible to substitute some almost equally serviceable drug for that asked for. I always made it my business to go to the supply depots and obtain what I required in person, and frequently I came back to my hospital with a far better supply than I had set out to obtain. Alchohol, whisky, brandy and gin could always be had in sufficient quantities, and I think that a quarter of a ship-load of castor oil was sent to Siboney. Nearly all drugs were put up in tablet form, which was very convenient for dispensing. Of course it was necessary to crush these tablets before administering them. Certain set formulas in tablet form, like the camphor opium pill and the carminative pill, were used too freely and to the positive injury of a great number of men. It always seemed more rational to me to relieve the cause of the diarrhea and dysenteries by a good free purge or irrigation of the bowels rather than to relieve temporarily the pain by the use of an opiate.

All cases of intestinal disturbances and fever were kept on a liquid diet.

Diarrheas were purged and then given intestinal antiseptics, carminatives and astringents.

Dysentery was treated in very much the same manner, except that I used ipecac, with marked success, in large doses of the powder or fluid extract, and irrigated the large bowel with two or three gallons of warm sterilized solution of permanganate of potash, 1 to 2,000, two or three times a day, or a solution of nitrate of silver was used. In fact, the general lines of treatment followed by me were similar to those usually practiced. In regard to yellow fever, I followed an expectant plan, meeting conditions as they arose, used intestinal antiseptics and a starvation diet. This latter was not difficult to impose.

Simple uncomplicated attacks of any given disease were exceedingly rare. The complicating diseases were intercurrent in most instances; frequently they superseded the original attack, and often followed closely on the establishment of convalescence. A man once taken sick never seemed to be able to get well, but went from one disease to another, each succeeding attack rendering him less able to recuperate or to withstand his next disease. From being a body of men having the highest morale and tone, our soldiers, through the influence of neglect, the climate and disease, became depressed and despondent, and many of them seemed to have only the purely animal instinct left, and the regulation of their bowels became almost a monomania; it was rare to find a man who did not wish to have his bowels either moved or checked.

In the fatal cases I held post-mortems, confirming my diagnosis of yellow fever, and ofttimes found serious complications, although, strange to say, I never found an abscess of the liver.

Malaria complicated, probably, 90 per cent. of the cases, and an equal percentage had either loose bowels or well-marked diarrhea or dysentery. The following is a crude classification of some 265 cases treated by me. I treated many more, but these will serve to show the variety of diseases and mortality:

| | Cases. | Deaths. | Percentage of Mortality. |
|---|---|---|---|
| Yellow fever | 185 | 20 | 10.8 |
| Yellow fever complicated by typhoid and malaria | 4 | 2 | 50 |
| Typhoid fever | 10 | 2 | 20 |
| Dysentery | 15 | .. | ... |
| Diarrhea | 10 | .. | ... |
| Appendicitis | 1 | .. | Operation. |
| Rheumatism, acute | 5 | .. | ... |
| Malaria, uncomplicated | 35 | .. | ... |

Eight cases of yellow fever died on day of admission. Three days before I left Cuba I procured a microscope and was able to examine the blood and urine of forty miscellaneous cases. In every instance I found the malarial organisms, those of the intermittent type being most numerous. In six or seven cases of yellow fever I found the estivo-autumnal form, and I was able in two fatal cases of typhoid fever complicated with yellow fever to find malarial organisms in the blood before death and at the post-mortems the characteristic lesions of typhoid fever and yellow fever.—*N. O. Medical and Surgical Journal.*

